# Assessment of *ERBB2 *and *EGFR *gene amplification and protein expression in gastric carcinoma by immunohistochemistry and fluorescence in situ hybridization

**DOI:** 10.1186/1755-8166-4-14

**Published:** 2011-06-20

**Authors:** Wang YK, Gao CF, Yun T, Chen Z, Zhang XW, Lv XX, Meng NL, Zhao WZ

**Affiliations:** 1 Department of Pathology, The 150 Centre Hospital of Chinese PLA, Medical Center of Jinan Military Region, Luoyang 471031, China

## Abstract

**Background:**

The goal of this study was to investigate *ERBB2(HER2) *and *EGFR *gene amplification and protein expression in gastric cancer. Fluorescence *in situ *hybridization (FISH) and immunohistochemistry were used to analyze *ERBB2 *and *EGFR *gene amplification and protein expression in 69 cases of gastric cancer.

**Results:**

FISH analysis revealed that 20.3% of the cases exhibited *ERBB2 *gene amplification. Increases in *ERBB2 *copy number and gene amplification were present in 52.2% of the samples. Expression of the *ERBB2 *protein was observed in 42.0% of cases. FISH analysis detected *EGFR *gene amplification in 29.0% of samples. Increases in *EGFR *copy number and gene amplification occurred in 57.9% of samples, and *EGFR *protein expression was present in 52.2% of samples. Both *ERBB2 *and *EGFR *gene amplification were 3 cases (4.3%), but abnormalities in both *ERBB2 *and *EGFR *gene copy number were present 36.2% of samples. *ERBB2 *and *EGFR *gene amplification were significantly associated with the depth of tumor invasion (*P *< 0.05) and lymph node metastasis (*P *< 0.05), but not with sex, age, or histological type (*P *> 0.05).

**Conclusions:**

Our data indicated that *ERBB2 *and *EGFR *genetic abnormalities were associated with the prognosis of gastric cancer. Clinical assessment of *ERBB2 *and *EGFR *amplification may represent an important factor for the development of personalized treatment programs for gastic cancer.

## Introduction

Overexpression of the human epidermal growth factor receptor 2 (*ERBB2*(*HER2*)), also known as C-erbB-2, can lead to the activation of cellular signal transduction systems, resulting in the cellular transformation and cell proliferation events associated with cancer [1]. ERBB2 is very similar in structure to the epidermal growth factor receptor (*EGFR*), with approximately 95% homology between the 260 amino acid intracellular region (aa 727-986), which contains the *EGFR *tyrosine kinase domain, and the corresponding domain in *EGFR*. Both *ERBB2 *and *EGFR *are membrane-associated tyrosine kinases and contain three functional domains: the extracellular ligand binding domain (aa 1-632), a lipophilic transmembrane segment (aa 633-654), and a cytoplasmic domain that exhibits tyrosine kinase activity (aa 655-1234) [2,3], *ERBB2 *is frequently unregulated in human cancers such as breast cancer [4], ovarian cancer [5], and so on. High expression level of *ERBB2 *has been significantly correlated with increased tumor invasion, metastasis, resistance to chemotherapy, and poor prognosis of patients [6]. However, a lack of reliable and accurate methods to assess the relationship between *EGFR *and *ERBB2 *gene status and protein expression in gastric cancer has limited correlative assessments with clinical parameters, including survival and sensitivity to targeted agents. Therefore, in the present study, we utilized fluorescence *in situ *hybridization (FISH) to assess amplification of the *ERBB2 *and *EGFR *genes in gastric cancer patient samples. We combined this data with results from histopathological and immunohistochemical analyses to determine the relationship between *ERBB2 *and *EGFR *expression status and clinicopathological variables in gastric cancer.

## Materials and methods

### Patients and tumor samples

A total of 69 gastric cancer patients who underwent radical gastrectomy at the Liberation Army No. 150 Central Hospital between July, 2008 and March, 2010 were included in this study. Tumor subtypes included 16 papillary adenocarcinomas, 14 tubular gland cancers, 15 mucinous adenocarcinomas, 13 poorly differentiated adenocarcinomas, and 11 signet ring cell carcinomas. The average age of the patients at the time of surgery was 59.2 years (range, 31-76 years). Tumor specimens were collected after obtaining informed consent from the patients in accordance with institutional guidelines. The samples were fixed in 10% neutral formalin in preparation for further studies.

### FISH analysis

*ERBB2 *and *EGFR *gene amplification were analyzed by FISH using the Vysis Path Vysion *ERBB2*/DNA probe kit and the LSI *EGFR *SpectrumOrange/*CEP 7 *SpectrumGreen probe, respectively, according to manufacturer's instructions (Abbott Molecular, Abbott Park, IL, USA). Briefly, samples fixed in 10% formalin and embedded in paraffin were cut into 4 μm sections and incubated overnight at 56 °C. Slides were dewaxed in xylene and dehydrated in 100% alcohol for 5 min, followed by air drying. The slides were then incubated in proteinase K solution (0.2 mg/mL in 2 × SSC) at 37 °C for 15 min, washed with 2 × SSC (pH 7.0), and sequentially dehydrated in 70%, 85%, and 100% ethanol. After the application of 10 μl of probe to the target area of the slide, a coverslip was placed over the sample and sealed. Following denaturation at 73 °C for 5 min, the slides were allowed to hybridize overnight at 37 °C in a humidified chamber. After hybridization, the slides were washed in 0.4 × SSC containing 0.3% Nonidet P40 at 65 °C for 2 min and rinsed twice in 2 × SSC containing 0.1% Nonidet P40 for 2 min. The slides were then immersed in 70% ethanol for 3 min and dried at room temperature. After the slides were counterstained with 4', 6-Diamidino-2-phenylindole dihydrochloride (DAPI), they were observed under a fluorescence microscope (Carl Zeiss, Goettingen, Germany)[7].

*ERBB2 *levels were reported as *ERBB2 *gene: *CEP 17 *ratios in order to normalize values to the total number of chromosomes within each cell. Amplification was defined as an *ERBB2 *gene/*CEP 17 *ratios greater than or equal to 2.0 [8]. *EGFR *gene amplification was defined based on the criteria suggested by M Varella-Garcia, et al [9]. Several FISH patterns were identified for *EGFR*: (1)*EGFR *FISH-positive: (a) at least 15 copies of the *EGFR *signals in ≥ 10% of tumour cells (b) Specimens that have ≥ 40% of cells displaying ≥ 4 copies of the *EGFR *signal.(c) *EGFR/CEP7 *ratio ~ 1, but the presence of gene cluster (≥ 4 spots) in ≥ 10% of tumour cells;(d) *EGFR/CEP7 *ratio ≥ 2 and the presence of gene cluster (≥ 4 spots) in ≥ 10% of tumour cells;(2) *EGFR *FISH-negative: Specimens that do not display gene amplification according to the criteria defined above and with <40% of cells displaying ≥ 4 copies of the *EGFR *signal.

### Immunohistochemistry

*ERBB2 *and *EGFR *protein expression were evaluated by immunohistochemistry using the HercepTest™ and *EGFR *pharmDx™ kits, respectively, according to the manufacturer's recommended protocols (DAKO, Carpinteria, CA, USA). Two pathologists independently scored slides as 0, 1^+^, 2^+^, or 3^+ ^according to the guidelines provided by DAKO. As defined in the manufacturer's instructions, scores of 0 or 1^+ ^were considered negative, a score of 2^+ ^was weakly positive, and a score of 3^+ ^was strongly positive.

### Statistical analysis

The SPSS 13.0 statistical package (SPSS, Inc., Chicago, IL, USA) was used for all statistical analyses. A χ^2 ^test was used for comparisons with *ERBB2 *and *EGFR *gene amplification. *P*-values less than 0.05 were considered to be statistically significant.

## Results

### *ERBB2 *gene amplification and protein expression

**Figure 1:**
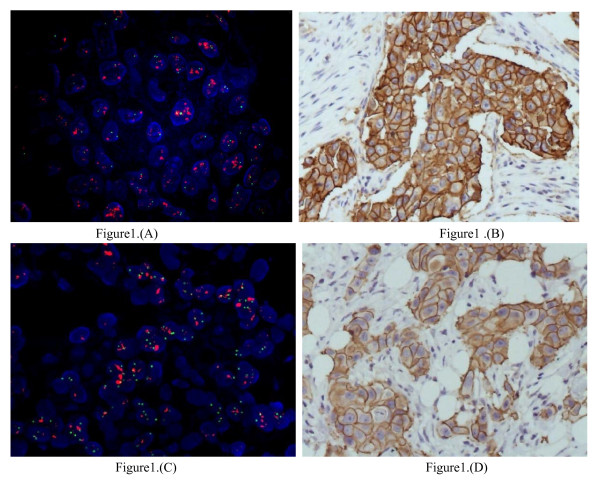
***ERBB2 *and *EGFR *gene amplification and protein expression in gastric cancer samples**. (A) Representative image showing *ERBB2 *gene amplification in a gastric adenocarcinoma sample analyzed by FISH. The ratio of red to green signal is greater than 2, indicative of *ERBB2 *gene amplification (magnification, 1000 ×). (B) Representative image showing *ERBB2 *protein expression analyzed by IHC in a poorly-differentiated gastric adenocarcinoma (IHC score, 3^+^; magnification, 200 ×). (C) Representative image showing *EGFR *gene amplification in a gastric adenocarcinoma sample analyzed by FISH. The green signal corresponds to chromosome 7, and the red signal corresponds to *EGFR*. *EGFR *gene amplification is visible as red clustering (magnification, 1000 ×). (D) Representative image showing *EGFR *protein expression analyzed by IHC in a poorly-differentiated gastric adenocarcinoma (IHC score, 3^+^; magnification, 200 ×).

Of the 69 gastric tumors, 14 (20.3%) cases exhibited *ERBB2 *gene amplification (Figure 1A). Of the remaining 55 cases without *ERBB2 *gene amplification, the copy number was increased in 22 cases. Therefore, *ERBB2 *copy number increases or gene amplification were observed in a total of 52.2% (36/69) of the samples. The *ERBB2 *protein was expressed (Figure 1B) in 42.0% (29/69) of the samples. Overexpression of the *ERBB2 *protein, evident as immunostaining scores of 2^+ ^or 3^+^, was frequently associated with *ERBB2 *gene amplification, as *ERBB2 *gene amplification was present in 7 of 9 tumors with a score of 2^+ ^and in 4 of 4 tumors with a score of 3^+^. Tumors that exhibited a staining score of 1^+ ^were classified as having a low amplification rate, as only 3 of 16 samples exhibited *ERBB2 *amplification. Samples with scores of 1^+ ^exhibited a significant difference in levels of *ERBB2 *amplification in comparison to samples with scores of 2^+ ^and 3^+ ^(*P *< 0.05, Table 1).

**Table 1:** Comparison of *ERBB2 *gene amplification and protein expression in 69 cases of gastric cancer

IHC score	IHC n (%)	FISH n (%)	χ2	*P-*value
0	40(57.9%)	55(82.7%)		
1^+^	16(23.3%)	3(4.3%)*	12.461	0.000
2^+^	9(13.0%)	7(10.1%)		
3^+^	4(5.8%)	4(5.8%)		
Total positive cases	29(42.0%)	14(20.3%)		

* In comparison to FISH results from combined samples with scores of 2^+ ^and 3^+^. IHC, immunohistochemistry; FISH, fluorescence *in situ *hybridization; n, number of positive samples.

### *EGFR *gene amplification and protein expression

Of the 69 patient samples, 29.0% (20/69) exhibited *EGFR *gene amplification (Figure 1C). Interestingly, overexpression of the *EGFR *protein in the absence of *EGFR *gene amplification was observed more frequently than expected. A total of 40/69 cases exhibited increases in *EGFR *gene copy number in the all samples. The *EGFR *protein was expressed in 52.2% (36/69) of patient samples (Figure 1D). Immunohistochemical analysis revealed that samples with *EGFR *protein expression scores of 3^+ ^and 2^+ ^exhibited significantly higher levels of gene amplification than those with scores of 1^+ ^(5/5 and 10/11 vs. 5/20, respectively; *P *< 0.05; Table 2).

**Table 2:** Comparison of *EGFR *gene amplification and protein expression in 69 cases of gastric cancer

IHC score	IHC n (%)	FISH n (%)	χ2	*P-*value
0	33(47.8%)	49(71.0%)		
1^+^	20(29.0%)	5(7.2%)*	17.016	0.000
2^+^	11(15.9%)	10(14.5%)		
3^+^	5(7.3%)	5(7.2%)		
Total positive cases	36(52.2%)	20(29.0%)		

* In comparison to FISH results from combined samples with scores of 2^+ ^and 3^+^. IHC, immunohistochemistry; FISH, fluorescence *in situ *hybridization; n, number of positive samples.

### Relationship between *ERBB2 *and *EGFR *gene abnormalities in gastric cancer

*ERBB2 *copy number alterations and gene amplification occurred in a total of 52.2% (36/69) of patient samples. *EGFR *copy number alterations and gene amplification occurred in a total of 57.9% (40/69) of patient samples. Both *ERBB2 *and *EGFR *gene amplification were 3 cases (4.3%), but abnormalities in both *ERBB2 *and *EGFR *gene copy number were present 36.2% of samples, indicating that abnormalities in the two genes may be correlated.

### Correlation between *ERBB2 *and *EGFR *gene amplification and clinicopatholigical parameters in gastric cancer

*ERBB2 *and *EGFR *gene amplification were not associated with sex, age, or histological type (*P *> 0.05) in gastric cancer samples. However, amplification of both *ERBB2 *and *EGFR *were significantly associated with the depth of invasion of the tumor and with lymph node metastasis. In our search, 24.4% (10/41) and 34.1% (14/41) of patients with lymph node metastases harbored *ERBB2 *and *EGFR *gene amplification, respectively. (*P *< 0.05, Table 3).

**Table 3:** Association of *ERBB2 *and *EGFR *gene amplification with clinicopathological parameters in 69 cases of gastric cancer

Parameter	n*	*ERBB2* Amp. * Positive (%)	χ^2 ^	*P-*value	*EGFR* Amp. * Positive (%)	χ^2 ^	*P-*value
Sex							
Male	41	8(19.5)	0.316	0.574	11(26.8)	0.261	0.609
Female	28	4(14.3)			6(21.4)		
Age							
≥ 60	36	7(19.4)	0.221	0.638	10(27.8)	0.400	0.527
< 60	33	5(15.2)			7(21.2)		
Histological type							
Papillary adenocarcinoma	16	2(12.5)			3(18.8)		
Tubular adenocarcinoma	14	2(14.3)			3(21.4)		
Mucinous adenocarcinoma	15	3(20.0)	0.729	0.948	4(26.7)	0.714	0.950
Poorly differentiated adenocarcinoma	13	3(23.1)			4(30.8)		
Signet ring cell carcinoma	11	2(18.2)			3(27.3)		
Gastric cancer staging							
T1/T2	25	1(4.0)	4.894	0.027	2(7.7)	5.845	0.016
T3/T4	44	11(25.0)			15(34.1)		
Lymph node metastasis							
Present	41	10(24.4)	6.264	0.012	14(34.1)	4.920	0.027
Absent	28	2(7.1)			3(10.7)		

* n, number of samples; Amp., amplification.

## Discussion

*EGFR *belongs to a family of four related receptors that includes the *EGFR *(*HER1*), *ERBB2*, *HER3*, and *HER4 *receptors [10]. The *EGFR *gene is located in the short arm of human chromosome 7 and produces a glycoprotein with a molecular weight of 170 kDa with high affinity for the *EGF *ligand [11]. *EGFR *mediates multiple signal transduction pathways and, thereby, connects extracellular signaling to intracellular changes in gene expression that modulate cellular growth and differentiation. Currently, *EGFR *and *ERBB2 *are the best characterized of the *HER *family receptors. However, little is known regarding the expression and function of these receptors in gastric cancer.

In recent years, interest has grown in understanding the relationship between the biological characteristics of gastric cancer and the association of these characteristics with the clinical outcomes of the disease. Current studies have focused on understanding the molecular basis of gastric cancer in order to help achieve accurate diagnoses and to better choose effective treatment options. With the advent of novel targeted therapeutics, these molecular characteristics may be important for obtaining more effective therapeutic outcomes. Presently, much of the available knowledge regarding *EGFR *and *ERBB2 *expression as well as their biological function in gastric cancer has come from countries other than China. Research techniques for characterization of *EGFR *and *ERBB2 *expression have included immunohistochemistry (IHC), chromogenic *in situ *hybridization (CISH), and FISH. Using kits approved by the United States Federal Drug Administration for IHC (HercepTest™ and *EGFR *pharmDx™) and FISH (Path Vysion™), we have analyzed *EGFR *and *ERBB2 *protein expression and gene status in Chinese gastric cancer patients in order to provide more accurate reference data for future studies in the Chinese population.

*EGFR *and *ERBB2 *expression in gastric cancer has been reported in many past studies. IHC was first used in 1986 to detect *ERBB2 *expression in gastric cancer [12], which was followed by a large number of similar reports. Reported rates of *ERBB2 *gene expression in gastric cancer range were from 9% to 38%. Moreover, the correlation between *EGFR/ERBB*2 expression in gastric cancer and prognosis remains controversial. In the present study, the *ERBB2 *protein was expressed in 42.0% (29/69) of gastric tumors, and *ERBB2 *gene amplification occurred in 20.3% (14/69) of tumors. Of 41 patients with lymph node metastases, *ERBB2 *gene amplification was present in 24.4% (10/41) of the cases. The *EGFR *protein was expressed in 52.2% (36/69) of gastric tumors, and *EGFR *gene amplification occurred in 29.0% (20/69) of tumors. Of patients with lymph node metastases, *EGFR *gene amplification was present in 34.1% (14/41) of the cases. These results suggest that *ERBB2 *and *EGFR *gene amplification are positively associated with *ERBB2 *and *EGFR *protein expression. Furthermore, a higher frequency of *ERBB2 *and *EGFR *gene amplification is present in gastric cancer patients with lymph node metastases. *EGFR *and *ERBB2 *expression in gastric epithelial cells are indicators of malignancy and may prove useful as markers for poor prognosis in gastric cancer.

At present, pathological examination is primary method used to assess the *ERBB2 *and *EGFR *status of tumor cells. In particular, IHC and FISH are commonly used in clinical settings. IHC is currently the most widely-used method, and kits are commercially-available for semi-quantitative detection of both *ERBB2 *and *EGFR*. Importantly, the higher levels of *ERBB2 *expression are associated with improved benefit of *ERBB2*-targeted anticancer drugs [13]. Application of IHC to evaluate the expression of *ERBB2 *in tumor cells has limitations, as the determination of staining is partially subjective, as opposed to strictly quantitative. In contrast, results from the FISH are substantially less subjective than IHC, while maintaining sensitivity and specificity. Furthermore, the quantitative nature of FISH results can effectively reduce operator interference and inter-laboratory variations [14]. In the present study, FISH revealed that *ERBB2 *gene amplification was present in 4 of 4 cases with an IHC score of 3^+^, in 7 of 9 cases with an IHC score of 2^+^, and in only 3 of 16 cases with an IHC score of 1^+^. These results indicated that a statistically significant difference in *ERBB2 *gene amplification was present between the high (2^+ ^and 3^+^) and low (1^+^) IHC score groups (*P *< 0.05). Similar results were observed with respect to *EGFR *gene amplification. *EGFR *gene amplification was observed in 5 of 5 samples with an IHC score of 3^+^, in 10 of 11 samples with a score of 2^+^, and in 5 of 20 samples with a score of 1^+^, which represented a significant difference between the high (2^+ ^and 3^+^) and low (1^+^) scores (*P *< 0.05). Based on these results, the FISH assay could prove to be truly valuable for determination of *ERBB2 *and *EGFR *expression in clinical practice.

In recent years, new developments in cancer biology have led to the emergence of novel molecular-targeted therapeutics. These targeted drugs selectively act on cancer cells at the molecular, biochemical, and genetic levels, specifically targeting abnormal cells, with minimal effects on the function of normal cells. For example, *EGFR *inhibitors have been used to block *EGFR *activity and, thereby, increase the radiosensitivity of tumor cells [15]. This phenomenon can lead to improvement of the efficacy of radiotherapy, likely because inhibition of *EGFR *signaling in tumor cells decreases cell proliferation, accelerates apoptosis, interferes with the cell cycle, and extends the time required for DNA repair after radiation [16]. In the present study, FISH analysis revealed that *EGFR *gene amplification was present in 24.6% of the cases analyzed, and IHC showed that the *EGFR *protein was expressed in 52.2% of the cases. These results suggest that in some radiation-resistant gastric cancer cases, targeting of *EGFR *for radiation sensitization therapy may have important clinical value.

Co-expression of *ERBB2 *and *EGFR *may have synergistic effects on the progression of gastric cancer [17]. *ERBB2 *gene amplification was observed in20.3% (14/69) of gastric cancer patients, including 24.4% (10/41) of the 41 patients with lymph node metastases and only 7.1% (2/28) of patients without lymph node metastases, representing a significant difference (*P *< 0.05). *EGFR *gene amplification was present in 29.0% (20/69) of gastric cancers, including 34.1% (14/41) of patients with lymph node metastases and 10.7% (3/28) of patients without lymph node metastases, again representing a significant difference (*P *< 0.05). *ERBB2 *and *EGFR *gene amplification were also significantly related. Increased *ERBB2 *copy number and gene amplification were present in a total of 57.9% (40/69) of samples. However, Both *ERBB2 *and *EGFR *gene amplification of gastric tumors were 3 cases (4.3%), but abnormalities both *ERBB2 *and *EGFR *gene copy number were present in 36.2% of samples. These results suggest that alterations in *ERBB2 *or *EGFR *gene status in gastric cancer are common events that frequently occur within the same tumor.

In summary, *ERBB2 *and *EGFR *are transmembrane tyrosine kinases that can promote tumorigenesis and tumor progression. Expression of *ERBB2 *and *EGFR *appear to be closely related, and one or both proteins are frequently overexpressed in gastric epithelial cancer cells. Furthermore, the degree of expression is correlated with tumor invasion, progression, and patient survival, suggesting that these genes may represent important indicators of poor prognosis. *EGFR *overexpression in gastric cancer commonly leads to radiation resistance. Therefore, *EGFR*-targeted radiosensitization treatments may have important clinical value for treatment of gastric cancer.
